# Exercise Intervention and Hospital-Associated Disability: A Nonrandomized Controlled Clinical Trial

**DOI:** 10.1001/jamanetworkopen.2023.55103

**Published:** 2024-02-08

**Authors:** Carlos Rodriguez-Lopez, Jennifer Mayordomo-Cava, Teresa Zarralanga-Lasobras, Vicente Romero-Estarlich, Maria Teresa Vidan, Javier Ortiz-Alonso, Pedro L. Valenzuela, Gabriel Rodriguez-Romo, Alejandro Lucia, Jose Antonio Serra-Rexach

**Affiliations:** 1 Centro de Investigación Biomédica en Red de Fragilidad y Envejecimiento Saludable, Instituto de Salud Carlos III, Madrid, Spain; 2 Department of Geriatrics, Hospital General Universitario Gregorio Marañón, Health Research Institute Gregorio Marañón, Madrid, Spain; 3 School of Medicine, Department of Medicine, Universidad Complutense, Madrid, Spain; 4 Physical Activity and Health Research Group, Research Institute of Hospital ‘12 de Octubre,’ Madrid, Spain; 5 Department of Systems Biology, University of Alcalá, Madrid, Spain; 6 Faculty of Physical Activity and Sport Sciences, Universidad Politécnica de Madrid, Madrid, Spain; 7 Faculty of Sport Sciences, Universidad Europea de Madrid, Madrid, Spain

## Abstract

**Importance:**

Inpatient exercise interventions may prevent, at least partly, hospital-associated disability (HAD) in older adults, but whether they also confer clinical benefits in the months following discharge is unclear.

**Objective:**

To examine the association of exercise and health education with HAD incidence in hospitalized older adults receiving acute hospital care at discharge and 3 months later.

**Design, Setting, and Participants:**

This single-center open-label, nonrandomized controlled clinical trial included patients aged 75 years or older seen at an acute care for elders unit at a tertiary public hospital in Madrid, Spain, from May 1, 2018, to June 30, 2022.

**Interventions:**

Patients were allocated to an intervention or control group. Both groups received usual care, but the intervention group also performed a supervised multicomponent exercise program (daily strength, balance, and walking exercises along with inspiratory muscle training) during hospitalization and received health education on how to exercise at home and telephone counseling during follow-up.

**Main Outcomes and Measures:**

The primary outcome was HAD incidence (determined by the Katz Index of Independence in Activities of Daily Living [hereafter, Katz Index]) at discharge and after 3 months compared with baseline (ie, 2 weeks before admission). Secondary outcomes included HAD incidence determined by the Barthel Index for Activities of Daily Living, ambulatory capacity decline at discharge and follow-up, changes in physical performance at discharge, and incidence of falls, readmissions, and mortality during the follow-up period.

**Results:**

The study included 260 patients (134 women [51.5%]; mean [SD] age, 87.4 [4.9] years [range, 75-105 years]; median hospital length of stay, 7 days [IQR, 5-10 days]), of whom 130 received the intervention and 130 were in the control group. Differences in HAD incidence did not reach statistical significance at discharge (odds ratio [OR], 0.62; 95% CI, 0.37-1.05; *P* = .08) or follow-up (OR, 0.65; 95% CI, 0.36-1.17; *P* = .15) when using the Katz Index. A lower HAD incidence was observed in the intervention group at discharge (OR, 0.47; 95% CI, 0.27-0.81; *P* = .01) and at follow-up (OR, 0.36; 95% CI, 0.20-0.66; *P* = .001) when using the Barthel Index for Activities of Daily Living. The intervention was also associated with a lesser decline in ambulatory capacity (OR, 0.55; 95% CI, 0.32-0.96; *P* = .03) and improved physical performance at discharge (Cohen *d*, 0.39; 95% CI, 0.12-0.65; *P* = .004). No significant associations were observed for readmissions, falls, or mortality.

**Conclusions and Relevance:**

In this nonrandomized controlled clinical trial, an exercise and health education intervention was not significantly associated with reduced HAD incidence when measured by the Katz Index. However, the benefits found for several secondary outcomes might support the implementation of in-hospital exercise programs for older patients.

**Trial Registration:**

ClinicalTrials.gov Identifier: NCT03604640

## Key Points

**Question:**

Is an in-hospital exercise and health education intervention associated with a reduced incidence of hospital-associated disability in older adults?

**Findings:**

In this nonrandomized controlled clinical trial of 260 patients, a multicomponent exercise program combined with health education was not associated with significant reductions in the incidence of hospital-associated disability in older patients receiving acute hospital care when assessed by the Katz Index of Independence in Activities of Daily Living.

**Meaning:**

These results suggest that incorporation of this intervention in the care plan of hospitalized elderly patients is not associated with a lower incidence of hospital-associated disability if it is only assessed by the Katz Index.

## Teaser

This nonrandomized controlled clinical trial assesses the association of a multicomponent exercise intervention combined with a health education program with the incidence of hospital-associated disability in hospitalized older patients.

## Introduction

Hospital-associated disability (HAD), commonly defined as a new loss of ability to perform 1 or more activities of daily living (ADLs) unaided following hospitalization,^1^ affects approximately 30%^2^ and approximately 50%^3^ of hospitalized adults receiving acute hospital care who are aged 70 years or older and 85 years or older, respectively. This condition is a major concern because it is associated with a high risk of hospital readmission, institutionalization, and mortality.^4,5^

Although loss of ADLs occurs mainly between the onset of acute illness and hospital admission,^3,5^ several factors associated with hospitalization (eg, mobility restriction, no self-care promotion, polypharmacy, delirium, and nosocomial infections) hinder the functional recovery of older patients at discharge.^6,7,8^ A meta-analysis^9^ reported that hospitalization in acute care for elders (ACE) units, which are specialized in providing care for geriatric syndromes while promoting functional independence through multidisciplinary teamwork, was associated with a reduced risk of HAD of 13% compared with hospitalization in general medicine or surgery units, with our ACE unit achieving a risk reduction of up to 19% in a previous study.^10^ However, further efforts are needed to prevent HAD, especially when considering that its prevalence has not decreased during the past 3 decades.^2,11^

Overall, the evidence suggests that physical exercise during hospitalization for acute care is safe and associated with reduced risk of HAD in older adults and with an improvement or slower decline in physical performance.^12^ Although interventions that focus solely on promoting mobility have shown beneficial associations with ADL function,^13,14^ multicomponent exercise programs (ie, combining different modalities, usually muscle-strengthening tasks together with aerobic or mobility and balance training) may provide additional benefits in terms of physical and cognitive performance.^15,16,17^ However, multicomponent training often relies on the use of specific equipment (eg, weights, gym machines), which may limit its implementation in hospital settings.^18^ In this context, a study by some of us previously showed that a simple exercise program in an ACE unit, consisting only of sit-to-stands and corridor walking, reduced the incidence of HAD at discharge in older adults (mean age, 88 years) compared with control individuals who did not receive this intervention.^19^ Yet, the intervention failed to provide clinical benefits 3 months later (ie, no change in the incidence of falls, readmission, or mortality),^19^ suggesting that adherence to exercise after discharge could maximize the beneficial effects of this type of intervention. In this regard, complementing an in-hospital exercise program with health education sessions may promote exercise adherence after discharge by improving patients’ self-efficacy.^20^

The main aim of this study was to assess the association of a multicomponent exercise intervention combined with a health education program with the incidence of HAD in older patients hospitalized in an ACE unit. Our primary hypothesis was that multicomponent exercise would be associated with reduced HAD at discharge, while health education would be associated with an extended benefit beyond discharge.

## Methods

### Study Design

We conducted a nonrandomized controlled clinical trial (NCT0360464) in compliance with the recommendations of the Consolidated Standards of Reporting Trials (CONSORT) statement.^21^ The study protocol was approved by the Comité de Ética de la Investigación con Medicamentos del Hospital General Universitario Gregorio Marañón (trial protocol in Supplement 1), and all procedures were conducted in accordance with the Declaration of Helsinki.^22^ All patients provided written informed consent before enrollment. Proxy consent was obtained from a relative when the patient was unable to provide written consent.

The study was conducted in the ACE unit of the Geriatric Department of the Gregorio Marañón Hospital (Madrid, Spain) from May 1, 2018, to June 30, 2022. All patients admitted to the unit (aged ≥75 years) during this period were screened for the following exclusion criteria: being unable to walk (ie, ≤2 points in the Functional Ambulatory Classification [FAC])^23^ or having severe disability (ie, ≤20 points on the Barthel Index for Activities of Daily Living [hereafter, Barthel Index])^24^ at baseline (ie, 2 weeks before admission, as assessed by retrospective interview); having unstable cardiovascular disease or any other major medical condition contraindicating exercise, including end-stage disease; having severe dementia (ie, ≥8 errors on the Pfeiffer test)^25^; being unable to communicate; having an expected length of stay of 48 hours or less; being transferred from another hospital unit; being transferred out of the ACE unit or having a planned admission elsewhere; and having previously participated in the study.

Eligible patients who agreed to participate were assigned to a control or intervention group in a time-dependent manner in blocks of 4 weeks (interspersed with a 1-week cessation period in recruitment to prevent the simultaneous presence of participants from both groups and to minimize between-group contamination). On average, the ACE unit admitted 800 patients per year during the study period. However, patient recruitment was temporarily interrupted during the COVID-19 pandemic (March 2020 to February 2021), and recruitment rates were also reduced during subsequent waves and holiday periods due to staff shortages.

### Intervention

Patients assigned to the control group received the usual care provided in the ACE unit, as detailed in the eMethods in Supplement 2 and elsewhere.^10^ In addition to usual care, participants assigned to the intervention performed an in-hospital, multicomponent exercise training program (combining muscle strengthening, balance, and walking exercises as well as inspiratory muscle training) twice daily (except weekends) that was supervised by a physiotherapist (T.Z.-L.) and a clinical exercise physiologist. The exercise program was complemented with daily in-hospital health education sessions and monthly telephone counseling after discharge aimed at maintaining the same exercise routine at home for 3 months after discharge (eMethods in Supplement 2; the Video provides a detailed description). Objectified or participant-reported adverse events were recorded by research staff (trial protocol in Supplement 1). A detailed description of costs and full-time equivalents assigned to the intervention is presented in the eMethods in Supplement 2.

**Video.** Description of the Exercise and Health Education Program Provided to Hospitalized Older Adults This video summarizes the multicomponent exercise session and health education that was conducted twice a day during hospitalization for older adults. The exercise sessions were composed of a mobility warm-up, upper and lower body strength exercises, balance exercise, aerobic exercise (walking), and inspiratory muscle training.

### Outcomes

#### Primary Outcome

We used the Katz Index of Independence in Activities of Daily Living [hereafter, Katz Index], which scores functional independence in 6 ADLs (eating, transferring from bed to chair, walking, using the toilet, bathing, and dressing), to assess HAD.^26^ Hospital-associated disability was defined as a loss of the ability to independently perform 1 or more ADLs at discharge (as determined by assessors) or at 3-month follow-up (as determined by telephone interview with patients or caregivers, as appropriate) compared with baseline (ie, 2 weeks before admission, as assessed by retrospective interview with patients or caregivers). Therefore, any reduction (by ≥1 points) in the Katz Index score compared with baseline was considered as HAD.

#### Secondary Outcomes

Because the Katz Index might be relatively insensitive to variations at low levels of ADL function,^27^ we also determined between-group differences in HAD incidence using the Barthel Index. The latter broadens the range of basic ADLs beyond the Katz Index by adding grooming, ambulation, stair climbing, and bowel and bladder control to the 6 aforementioned ADLs and provides a global score of 0 to 100 (with 0 indicating the worst ADL function and 100 indicating the best ADL function).^24^ Between-group differences were also assessed for reduced ambulatory capacity using the modified FAC score, which classifies individuals in 5 different categories according to the level of assistance needed during walking (from 0 points for those unable to walk to 4 points for those able to walk independently on level ground or stairs).^23^ Both the Barthel Index and the modified FAC were also determined at baseline (by retrospective interview with the patients [or caregivers, when needed]), discharge (by assessors), and 3-month follow-up (by telephone interview). Any reduction in the Barthel Index score (by ≥5 points) or the FAC score (by ≥1 points) compared with baseline confirmed the presence of HAD or reduced ambulatory capacity, respectively.

We assessed the change in physical performance from admission to hospital discharge using the Short Physical Performance Battery (SPPB)^28^ and the Alusti Test.^29^ The SPPB evaluates static balance, gait speed, and sit-to-stand performance, providing a score of 0 (worst) to 12 (best),^28^ whereas the Alusti Test measures a broader range of physical performance tasks in hospitalized older individuals, with a composite score of 0 (worst) to 100 (best) based on 10 different items (including joint mobility, transfer ability, stability, walking capacity, and balance).^29^ The Alusti Test has been shown to be valid, reliable, and sensitive for assessing institutionalized and hospitalized older people.^29,30^ We used telephone interviews with patients (or caregivers, when needed) to determine the incidence of falls, hospital readmission, and mortality during the 3-month follow-up period. Outcome assessment involved nurses and geriatricians who were not blinded to group allocation.

### Statistical Analysis

The sample size was determined based on a previous study by some of us.^19^ Thus, a sample size of 252 participants or more was estimated to detect an intervention effect that reduces the prevalence of HAD at 3 months after discharge from 60% to 40% (statistical power, 80%; 2-sided type I error rate, 5%; assuming a mortality rate of 12% and 20% of withdrawals).

Descriptive data are presented as mean (SD) or number (percentage). The primary analysis was conducted under the intention-to-treat principle (ie, including all participants regardless of their adherence to the intervention), following the CONSORT guideline. Between-group differences in the incidence of HAD and decline in ambulatory capacity were determined through unadjusted binary logistic regression. Unadjusted negative binomial regression analyses were conducted to compare the incidence of falls and hospital readmissions during the 3-month follow-up, with Cox proportional hazards regression used to compare mortality rates. Between-group comparisons of changes in physical performance during hospitalization were conducted using linear mixed models, with participants entered as random intercepts and group (intervention, control), time (admission, discharge), and group × time interaction as fixed effects; Bonferroni corrections were applied for post hoc analyses.

Additional ad hoc analyses were conducted to enhance the statistical power and precision of treatment effect estimates. Accordingly, multiple imputation was used to address missing data in primary and secondary outcomes (eMethods in Supplement 2). The same statistical tests used in the primary analysis were also performed on imputed data, and additional analyses were performed using the baseline value of the relevant outcome as a covariate except for falls and readmission rates. Since linear mixed models are considered robust to missing data,^31^ physical performance outcomes (ie, SPPB and Alusti Test) were not included in the ad hoc analysis. Additionally, per protocol analyses were performed for patients adhering to the exercise program (ie, completing ≥50% of the prescribed in-hospital and home exercise sessions) following the same statistical procedures as for the primary analysis. All statistical analyses were done with SPSS, version 24.0 (IBM), with 2-sided α = .05.

## Results

Of 1293 patients screened, a total of 260 (20.1%) eligible patients (126 men 48.5%]; 134 women [51.5%]; mean [SD] age, 87.4 [4.9] years [range, 75-105 years]; median length of stay, 7 [IQR, 5-10] days) volunteered to participate in the study (130 per group) (Figure). Baseline characteristics by group are shown in Table 1.^32,33,34^ The intervention group had more men, more patients with a history of cancer, and better nutritional status at the time of admission. No other clinically meaningful differences between groups were observed.

**Figure.**
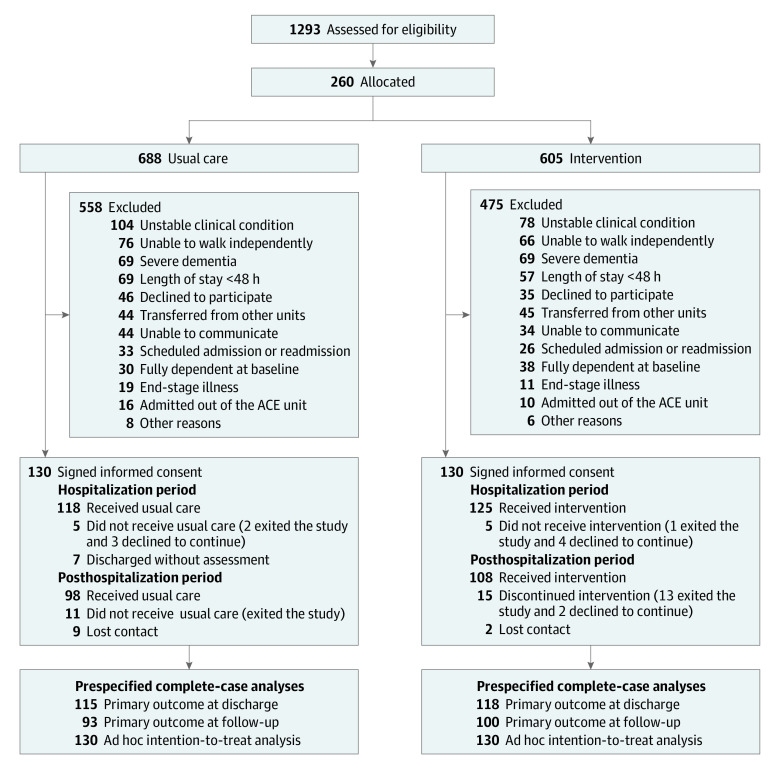
Study Flow Diagram ACE indicates acute care for elders.

**Table 1.** Participants’ Characteristics at the Start of the Study and Length of Hospitalization by Group

Variable	Participants^a^	
	Intervention (n = 130)	Control (n = 130)
Age, mean (SD), y	87.4 (4.9)	87.5 (5.0)
Sex		
Female	58 (44.6)	76 (58.5)
Male	72 (55.4)	54 (41.5)
BMI, mean (SD) ^b^	27 (4.3)	26.0 (4.5)
Living at home^c^	123 (95.3)	123 (95.3)
Comorbidities		
High comorbidity^d^	75 (58.6)	71 (55.5)
Heart failure^b^	68 (53.1)	64 (50.0)
Diabetes^b^	47 (36.7)	44 (34.4)
Moderate-severe CKD^b^	39 (30.5)	36 (28.1)
COPD^b^	43 (33.6)	31 (24.2)
Stroke^b^	24 (18.8)	24 (18.8)
Myocardial infarction^b^	25 (19.5)	22 (17.2)
Cancer^b^	26 (20.3)	14 (10.9)
Dementia^b^	17 (13.3)	20 (15.6)
Geriatric syndromes		
Frailty phenotype^e^	72 (56.7)	80 (64.0)
Depression^f^	33 (26.4)	44 (34.1)
Falls^g^	35 (27.6)	41 (31.8)
Pressure ulcers^b^	3 (2.3)	3 (2.3)
Polypharmacy (≥7 drugs)^g^	99 (78.0)	94 (72.9)
MNA-SF screening score, mean (SD)^h^	11.1 (2.4)	10.3 (2.4)
Malnutrition risk	44 (37.0)	56 (49.1)
Confirmed malnutrition	10 (8.4)	18 (15.8)
Main admission diagnosis^i^		
Circulatory	29 (23.0)	36 (28.8)
Infection	26 (20.6)	24 (19.2)
Digestive	20 (15.9)	16 (12.8)
Respiratory	16 (12.7)	8 (6.4)
Blood or myeloproliferative disease	12 (9.5)	12 (9.6)
Functional capacity		
At baseline (2 wk before admission)		
Katz Index score, mean (SD)^j^	4.5 (1.7)	4.4 (1.7)
Independent ambulation^k^	95 (74.2)	88 (67.7)
At admission		
Katz Index, mean (SD)^l^	3.1 (2.0)	2.8 (1.9)
Independent ambulation^m^	57 (45.6)	50 (40.0)
Length of stay, median (IQR), d	7.0 (5.0)	6.0 (4.0)

Abbreviations: BMI, body mass index (calculated as weight in kilograms divided by height in meters squared); CKD, chronic kidney disease; COPD, chronic obstructive pulmonary disease; MNA-SF, Mini Nutritional Assessment Short Form.

^a^ Data are presented as number (percentage) of patients unless otherwise indicated.

^b^ Data missing for 4 patients (2 in each group).

^c^ Data missing for 2 patients (1 in each group).

^d^ Defined as having a Charlson Comorbidity Index score of 3 or higher.^32^

^e^ Defined as having 3 or more of 5 Fried criteria.^33^

^f^ Data missing for 6 patients (2 in the control group and 4 in the intervention group).

^g^ Data missing for 4 patients (1 in the control group and 3 in the intervention group).

^h^ Ranging from 0 (worse) to 14 (best)^34^; data missing for 27 patients (16 in the control group and 11 in the intervention group).

^i^ Data missing for 9 patients (5 in the control group and 4 in the intervention group).

^j^ Higher scores indicate better function; data missing for 2 patients in the intervention group.

^k^ Independent ambulation considered if the Functional Ambulatory Classification score was 4 or higher^23^; data missing for 2 patients in the intervention group.

^l^ Data missing for 10 patients (3 in the control group and 7 in the intervention group).

^m^ Data missing for 10 patients (5 in each group).

Participants in the intervention group performed a median of 5 (IQR, 3-7) in-hospital exercise sessions (median duration per session, 34 [IQR, 30-37] minutes). Adherence to the planned in-hospital sessions was 50 (17%), with 81 patients (75.0%) in the intervention group reporting having exercised at home 15 days or more per month during the following 3 months. Overall, 59 patients (54.6%) in the intervention group completed 50% or more of the scheduled sessions during hospitalization and reported having exercised 15 days or more per month thereafter (eTable 1 in Supplement 2 give further details). No major adverse events were registered during the exercise sessions performed in the hospital or during follow-up.

When HAD incidence was assessed by the Katz Index (primary outcome), the difference between the intervention and control did not reach statistical significance at discharge (odds ratio [OR], 0.62; 95% CI, 0.37-1.05; *P* = .08) or at 3-month follow-up (OR, 0.65; 95% CI, 0.36-1.17; *P* = .15) (Table 2). In ad hoc adjusted analyses, the difference was not significant at discharge (OR, 0.59; 95% CI, 0.35-1.00; *P* = .050) or at 3-month follow-up (OR, 0.62; 95% CI, 0.35-1.08; *P* = .09) (eTable 2 in Supplement 2). In contrast, when HAD incidence was determined using the Barthel Index (ie, secondary outcome), the intervention was associated with reduced HAD incidence at both discharge (OR, 0.47; 95% CI, 0.27-0.81; *P* = .01) and at 3-month follow-up (OR, 0.36; 95% 0.20-0.66; *P* = .001) (Table 2). Furthermore, the intervention was associated with a decrease in ambulatory capacity decline at discharge (OR, 0.55; 95% CI, 0.32-0.96; *P* = .03), albeit with no significant between-group differences observed at 3-month follow-up (OR, 0.60; 95% CI, 0.32-1.14; *P* = .12). Consistent results were found in the ad hoc analyses (eTable 2 in Supplement 2).Regarding physical performance measures, the intervention was associated with improved gait score SPPB subcomponent (intervention: mean, 0.3 [95% CI, 0.2-0.5]; control: mean, 0.1 [95% CI, −0.1 to 0.2]; *P* = .03), but no differences were found for the total score (intervention: mean, 0.6 [95% CI, 0.2-0.9]; control: mean, 0.3 [95% CI, 0-0.7]; Cohen *d*, 0.13 [95% CI, −0.12 to 0.38]; *P* = .32) or the rest of the subcomponents of the SPPB (Table 3). However, when physical performance was assessed with the Alusti Test, the intervention was associated with an improved global test score (intervention: mean, 4.2 [95% CI, 2.1-6.3]; control: mean −0.1 [95% CI, −2.1 to 1.9]; Cohen *d*, 0.39 [95% CI, 0.12-0.65]; *P* = .004) and gait capacity subdomain (intervention: mean, 2.5 [95% CI, 1.2-3.7]; control: mean, 0.4 [95% CI, −0.9 to 1.7]; *P* = .02). No other differences were noted for other subdomains. Finally, no between-group differences were noted for readmissions, falls, or mortality rates (Table 2), with ad hoc analyses confirming these results (eTable 2 in Supplement 2).

**Table 2.** Association of the Intervention With HAD, Ambulatory Capacity Decline, Falls, Readmission, and Mortality

Outcome	Patients, No./total No. (%)		Unadjusted estimate (95% CI)	*P* value
	Intervention	Control		
HAD determined by Katz Index				
At discharge	56/118 (47.5)	68/115 (59.1)	0.62 (0.37-1.05)^a^	.08
At 3-mo follow-up	32/100 (32.0)	39/93 (41.9)	0.65 (0.36-1.17)^a^	.15
HAD determined by Barthel Index				
At discharge	68/119 (57.1)	83/112 (74.1)	0.47 (0.27-0.81)^a^	.01
At 3-mo follow-up	27/96 (28.1)	48/92 (52.2)	0.36 (0.20-0.66)^a^	.001
Ambulatory capacity decline^b^				
At discharge	31/122 (25.4)	44/115 (38.3)	0.55 (0.32-0.96)^a^	.03
At 3-mo follow-up	23/103 (22.3)	30/93 (32.3)	0.60 (0.32-1.14)^a^	.12
Readmission^c^	25/103 (24.3)	30/93 (32.3)	0.64 (0.39-1.06)^d^	.08
Falls^c^	16/102 (15.7)	18/90 (20.0)	0.55 (0.27-1.28)^d^	.10
Mortality	14/130 (10.8)	13/130 (10.0)	1.03 (0.48-2.20)^e^	.94

Abbreviation: HAD, hospital-associated disability.

^a^ Odds ratio.

^b^ Ambulatory capacity decline considered in the event of a decrease in Functional Ambulatory Classification score.^22^

^c^ A total of 37 readmissions were registered for the intervention group and 51 in the control group, and a total of 18 and 29 falls were registered in the intervention and control groups, respectively. The follow-up was 3 months after hospitalization (median, 98 [IQR, 93-105] days).

^d^ Incidence rate ratio.

^e^ Hazard ratio.

**Table 3.** Association of the Intervention With Physical Performance After Hospitalization^a^

Score (range)	Mean (95% CI)				*P* value^b^
	Intervention		Control		
	Admission	Change at discharge	Admission	Change at discharge	
**SPPB** ^c^					
Total score (0-12)	4.5 (4.0 to 5.0)	0.6 (0.2 to 0.9)	3.7 (3.2 to 4.1)	0.3 (0 to 0.7)	.32
Balance score (0-4)	1.8 (1.5 to 2.0)	0.3 (0.1 to 0.5)	1.5 (1.3 to 1.7)	0.2 (−0.1 to 0.4)	.49
Gait score (0-4)	1.9 (1.7 to 2.1)	0.3 (0.2 to 0.5)	1.6 (1.4 to 1.8)	0.1 (−0.1 to 0.2)	.03
Gait speed, m × s^−1^	0.5 (0.5 to 0.6)	0.1 (0 to 0.1)	0.5 (0.4 to 0.5)	0 (0 to 0.1)	.07
5STS score (0-4)	0.9 (0.7 to 1.0)	0 (−0.2 to 0.2)	0.6 (0.5 to 0.8)	0 (−0.2 to 0.2)	.82
5STS performance, s	13.2 (9.0 to 17.4)	−0.8 (−6.6 to 4.9)	13.6 (9.4 to 17.7)	−2.0 (−7.8 to 3.8)	.77
**Alusti Test** ^d^					
Total score (0-100)	61.6 (59.1 to 64.1)	4.2 (2.1 to 6.3)	61.6 (59.1 to 64.0)	−0.1 (−2.1 to 1.9)	.004
Joint mobility (0-28)	25.2 (24.6 to 25.8)	0.3 (−0.3 to 0.9)	25.4 (24.8 to 26.0)	−0.3 (−0.9 to 0.3)	.16
Transfer and stability (0-20)	15.2 (14.4 to 16.0)	1.0 (0.3 to 1.8)	15.1 (14.3 to 15.9)	0.7 (0 to 1.5)	.55
Gait (0-32)	21.0 (19.7 to 22.4)	2.5 (1.2 to 3.7)	20.1 (18.8 to 21.5)	0.4 (−0.9 to 1.7)	.02
Balance (0-20)	2.1 (0.6 to 3.7)	0.2 (−1.6 to 2.0)	2.8 (1.3 to 4.3)	−1.0 (−3.1 to 1.1)	.39

Abbreviations: 5STS, 5 times sit-to-stand test; SPPB, Short Physical Performance Battery.

^a^ All data were derived from the linear mixed-effects model.

^b^ The association of the intervention with each outcome was determined with the time × group interaction *P* value.

^c^ A total of 255 patients were considered in the analysis of SPPB differences.

^d^ A total of 253 participants were considered in the analysis of Alusti Test differences.

Per protocol analyses also confirmed the abovementioned findings. However, in contrast to the primary analyses, a significantly lower incidence of HAD was observed at 3-month follow-up as determined by both the Katz Index and the Barthel Index, and an association between the intervention and ambulatory decline at 3 months was found (eTables 4 and 5 in Supplement 2).

## Discussion

The present study found that compared with the usual care provided in the ACE unit, an intervention combining multicomponent exercise and health education was not significantly associated with decreased occurrence of HAD in older patients, as determined by the Katz Index (primary outcome). However, when HAD was assessed using the more comprehensive Barthel Index, the intervention was associated with a reduced incidence of HAD not only at discharge but also at 3-month follow-up. Although there is no consensus on the best instrument for monitoring HAD in older patients, those that include a broader range of ADLs and assess level of independence, such as the Barthel Index, may be more sensitive to changes. Thus, it appears that multicomponent exercise may be associated with reduced HAD (at least if determined by the Barthel Index) at discharge and health education may extend the benefit beyond discharge, although only partially. Furthermore, the intervention was associated with reduced deterioration in ambulatory capacity and with increased physical performance, as assessed by the Alusti Test.

In terms of hospital readmissions, our results differ from those of Courtney et al,^35^ who reported that multicomponent exercise and telephone counseling reduced readmission rates and general practitioner visits in hospitalized older adults. However, in line with our findings, meta-analytic evidence overall has shown that exercise interventions are not associated with hospital readmissions, falls, or mortality,^12,36^ which might support the safety of these interventions. Nevertheless, larger studies with longer follow-up are needed to confirm these findings.

The intervention group had improved physical performance at discharge as assessed by the Alusti Test, whereas no between-group differences were found for the SPPB. This discrepancy could be explained, at least in part, by the differences between the 2 tests; the Alusti Test might remove the floor effect (overlooking subtle changes in physical function, such as being able to raise from a chair using an armrest or increasing walking endurance capacity) that characterizes the SPPB, making it more suitable for populations with poor physical performance (eg, hospitalized older people).^30^ On the other hand, our finding that the intervention was associated with improved Alusti Test scores at discharge compared with the control group is consistent with meta-analytic evidence supporting the benefits of structured physical exercise programs for ADL independence and physical performance in hospitalized older people receiving acute hospital care.^12^ Thus, our findings, taken together with previous evidence,^12,36^ suggest that implementing structured and, ideally, multicomponent exercise programs may be associated with reduced nosocomial disability in older adults during acute care hospitalization.

It can be hypothesized that the overall low adherence to our intervention, which fell within the lower limit of the broad range (ie, 50%-90%) reported in previous studies,^15,35,37,38^ may have masked the intervention’s potential benefits. As shown in the per protocol analyses, the effect estimates for the association between the intervention and HAD incidence and physical performance were larger when patients completed at least 50% of the scheduled exercise sessions. Accordingly, further studies should investigate barriers to (and enablers of) exercise adherence during and after hospitalization.^18,39^

### Strengths and Limitations

The current study has several strengths. The study sample included older people with a high prevalence of the frailty phenotype and a range of comorbidities. Additionally, the sample size could be considered large compared with previous research in the field.^12^ Moreover, the current exercise program was designed to be performed at the patients’ bedside and at home without the need for weight-based strength training material so that it could essentially be implemented in any setting. Finally, the intervention provided additional benefits to the standard care in the ACE unit, which already includes patient mobilization and delirium prevention programs to prevent physical and cognitive decline during hospitalization. Therefore, more beneficial intervention effects would be expected if a comparator group of older patients hospitalized outside the ACE unit were used.

This study also has some limitations. We could not ensure assessor blindness due to limited personnel resources, partly caused by the workload imposed by the COVID-19 pandemic and its subsequent waves during the study period, which necessitated a deviation from the original study protocol. Additionally, the reliance on patient-reported ADLs, although confirmed with caregivers, could also be considered a limitation. Although the sequential allocation of treatment every 4 weeks minimized between-group contamination bias, it also introduced a potential source of bias associated with a noncompletely random allocation. Moreover, although our exclusion criteria were not overly restrictive, only a small proportion of the patients admitted to the ACE unit were eligible for the study due to most of them having complex clinical conditions. This, together with the fact that this was a single-center study, limits the potential generalizability of our results.

## Conclusions

In this nonrandomized controlled clinical trial, a multicomponent exercise program combined with health education was not associated with reduced incidence of HAD in hospitalized older adults at discharge or in the following 3 months when using the Katz Index. However, the intervention was associated with reduced HAD incidence when using the Barthel Index and with a lesser decline in ambulatory capacity and improved physical performance at discharge. These findings advocate for considering such interventions in the care plan for older patients during and after hospitalization.

## Notes

Supplement 1.
Trial Protocol

Supplement 2. **eMethods.** Supplementary Methods **eTable 1.** Detailed Description of Exercise Adherence **eTable 2.** Ad Hoc Analysis With Multiple Imputation: Effects of the Intervention on Hospital-Associated Disability (HAD), Ambulatory Capacity Decline, Falls and Readmission Rates. **eTable 3.** Per Protocol Analysis of Participants’ Characteristics at the Start of the Study and Length of Hospitalization, by Group **eTable 4.** Per Protocol Analysis of the Effects of the Intervention on Hospital-Associated Disability (HAD), Ambulatory Capacity Decline, Falls and Readmission Rates **eTable 5.** Per Protocol Analysis of the Effects of the Intervention on Physical Performance After Hospitalization
eReferences

Supplement 3.
Data Sharing Statement
