# Supplementary material for: Exercise Intervention and Hospital-Associated Disability: A Nonrandomized Controlled Clinical Trial
Source: JAMA Netw Open. 2024 Feb 8;7(2):e2355103. doi: 10.1001/jamanetworkopen.2023.55103 (PMC10853827; doi:10.1001/jamanetworkopen.2023.55103)
Supplement: Supplement 1. — Trial Protocol [file jamanetwopen-e2355103-s001.pdf]

The following document is a faithful translation of the latest approved version of the study protocol by the ethics committee of Hospital General Universitario Gregorio Marañon on April 9th 2018, acta 07/18.

Title: Physical training and health education in hospital-associated functional decline in the oldest old – AGECAR Plus: study protocol for a randomized controlled trial.

PI: Dr. Jose Antonio Serra Rexach

ClinicalTrials.gov ID: NCT03604640

1. BACKGROUND

According to data from the National Institute of Statistics, Spain continues to experience an aging process. Currently, 18.4% of the population is over 65 years old, and the population segment that is growing the most is the group of individuals over 80 years old, which currently represents 6% of the total population and is expected to increase to 9% in the next 15 years (1). One of the factors that most influence successful and healthy aging is functional independence, understood as the ability to independently perform activities of daily living (ADL) (2).

Disability associated with hospitalization (DAH): Hospitalization often impairs the functional capacity of elderly individuals, especially the frailest ones (3-6). This DAH is due to multiple barriers to mobility during hospitalization related to the patient (disease, comorbidities, etc.), treatment (rest, procedures, medications, etc.), the institution (availability of personnel, assistive equipment, etc.), and the attitudes of the patient and staff (7). A recent study demonstrates that beyond individual risk factors, one of the main factors associated with DAH at discharge and 1 month later is low mobility during hospitalization (5). Ten days of bed rest cause a significant loss of total lean mass and strength in the lower limbs (8, 9). Muscle strength loss can be as high as 5% per day, so ten days of hospitalization can lead to a 50% loss of strength (5). Furthermore, the consequences of inactivity are more severe in the elderly compared to younger individuals (10). DAH is highly prevalent: between 40-50% of hospitalized elderly individuals, even for a short period of time, experience a decline in their functional capacity at the time of discharge (11-14). The progression of this decline once the acute illness that led to hospitalization has resolved is highly variable: between 30 and 60% of elderly individuals who experienced disability during the hospitalization recover it some time after discharge (15). Data collected in our acute care unit show that 389 out of 627 patients (62%) are discharged with DAH (data being prepared for publication). The consequences of disability are devastating for the patient and the healthcare system (5). Many patients who are discharged with new disabilities and do not recover are unable to live alone, requiring supervision at home or institutionalization into a residential facility. Additionally, DAH is associated with poor hospital outcomes: longer stays, increased complications, and increased mortality. Thus, elderly individuals who experience less functional deterioration during hospitalization have a 10.7% three-month mortality rate, compared to 36.7% for those who experience the greatest functional deterioration (16). Data from our service indicate an increase in mortality at 6 and 12 months for patients discharged with DAH, compared to patients who did not experience deterioration or recovered their functional status at discharge (data being prepared for publication). Typically, hospital stays do not promote mobility in the elderly. Between 73 and 83% of the time an elderly individual is hospitalized, they are usually bedridden (17, 18). Even very short hospital stays can involve bed rest and low levels of activity, which are associated with negative functional outcomes (6). Compared to patients with higher levels of activity during hospitalization, those with bed rest or low levels of any type of activity (physical therapy, independent or assisted activity) had higher rates of DAH and a greater risk of institutionalization and death at discharge and thirty days post-discharge (6, 19). This relationship between low mobility levels and functional decline remains significant after adjusting for multiple variables and cofactors, such as

disease severity (19). Therefore, preventing inactivity and loss of muscle strength during hospitalization is the best way to prevent DAH.

Intervention Studies: The first controlled clinical trials on the effectiveness of interdisciplinary geriatric acute care units designed to prevent or rehabilitate DAH have shown an improvement in functional status at hospital discharge (20, 21). Our interdisciplinary model in the acute care unit also demonstrates a benefit in reducing functional decline and the incidence of delirium during hospitalization (22). Various meta-analyses and systematic reviews demonstrate that interdisciplinary programs aimed at maintaining or improving the functional capacity of the elderly during hospitalization have the potential to improve functional status at discharge, as well as reduce mortality rates, length of stay, and institutionalization rates (23, 24). It seems that interventions that include a follow-up program can maintain positive effects for longer periods of time (24). These studies have limitations: they are very diverse in terms of the type of patients (young or very old elderly), lack a clear description of the type, frequency, and duration of rehabilitation program exercises. Additionally, due to being multidisciplinary programs focused not only on functionality, it is difficult to determine the isolated effect of exercise on functionality. A recent study explores the association of the different components of interdisciplinary models with functional improvement: medication review, early rehabilitation, and individualized treatment appear to be the most effective components (25). There are few studies on the effect of exercise separated from the other components of interdisciplinary models and show contradictory functional results (26, 27, 28).

AGECAR Study: Our group has worked on physical activity programs for both institutionalized (29) and hospitalized (30) elderly individuals. In the latter setting, in 2012, we designed a Controlled Clinical Trial of an Exercise Program in Frail Elderly Patients Hospitalized in an Acute Geriatric Unit (30). As a summary (data sent for publication), we included 268 patients, with a mean age of 88 years, 70% of whom met frailty criteria, admitted to an acute geriatric unit. They were randomly assigned, on a monthly basis to avoid contamination, to an intervention group (IG) and a control group (CG). The intervention consisted of an exercise program, in addition to the usual treatment of the control group. The key elements of the daily exercise program were lower limb strengthening through squats and walks of up to 10 minutes in the hallway. The usual treatment of the control group is aimed at preventing functional decline and delirium. The main outcome variable was the change in functional status (measured by scales of basic activities of daily living and ambulation) from admission to hospital discharge and 3 months after discharge. More patients in the IG, compared to the CG, improved from admission to discharge in the number of activities they performed independently, and fewer patients worsened. The proportion of patients who recovered their baseline activities of daily living at 3 months was slightly better in the IG, but the difference was not significant. No beneficial effect was found on the degree of ambulation independence at discharge or at 3 months. This randomized clinical trial provides evidence that an easily applicable exercise program can discreetly improve the ability of a heterogeneous group of elderly patients admitted with acute illness, many of whom are nonagenarians and frail, to independently perform activities of daily living at hospital discharge, but not at 3 months. One of the main limitations of our study was that the median number of days the participants in the IG exercised was only three days. Given the prognostic importance (vital and functional) of disability associated with hospitalization for acute illness, we consider it essential to emphasize exercise programs during hospitalization and health education that improve this disability. Therefore, we intend to introduce modifications to the original program that improve this prognosis and maintain it over time. Basically, the modifications would involve ensuring more days of exercise during admission and including health education sessions so that the patient continues to perform the activities learned in the hospital at home after discharge.

2. HYPOTHESIS

A physical training and health education program for elderly patients hospitalized in an acute care unit improves the recovery of disability associated with hospitalization at discharge, and this beneficial effect is maintained at 3 months.

3. OBJECTIVES

Primary Objective:

To investigate the effect of a physical training program conducted during hospitalization, consisting of gait stimulation, lower limb muscle strengthening, and health education, on the physical functional capacity of the elderly, as measured by the proportion of patients who recover their baseline Katz index (prior to acute illness) at 3 months post-hospitalization.

Secondary Objectives:

To evaluate the effect of the intervention on recovery at discharge and at 3 months post-hospitalization, in terms of functional status measured by other scales of physical function (Barthel Index) and ambulation (Functional Ambulation Classification, FAC).

To evaluate the effect of the intervention on the change from admission to discharge in functional status measured by the Short Physical Performance Battery (SPPB).

To evaluate the consistency of differences between the intervention and usual care in subgroups defined by age, frailty, presence of delirium, and baseline functional status.

4. DESIGN

Randomized clinical trial designed according to the Consolidated Standards of Reporting Trials statement (CONSORT) recommendations (Altman DG, Ann Intern Med 2001; 134:663-694). After signing informed consent, subjects will be randomly assigned, in blocks of four weeks, to the intervention group (IG) or the control group (CG). The IG will undergo the physical training and health education program, while the CG will receive standard hospital care. A follow-up phone call will be conducted at three months after discharge to assess mortality, number of falls, and functional status. All tests (at admission and at discharge) will be conducted in the same hospital unit by the same evaluators not involved in the intervention. The study will be conducted following the Ethical Guidelines of the Helsinki Declaration.

SUBJECTS

Subjects admitted to the Acute Care Unit of the Geriatrics Department at Gregorio Marañón General University Hospital in Madrid. Initial screening will be performed within the first 72 hours of admission. Inclusion criteria will be age over 74 years, ability to walk (with or without assistance) before admission, ability to communicate, understand, and sign informed consent. Exclusion criteria will be hospital stay duration less than 72 hours, presence of any factor that hinders program participation, including terminal illness, myocardial infarction or limb fracture in the previous three months, inability to ambulate, severe dementia. If a patient who has already been included is readmitted later, they will not be included again.

RANDOMIZATION

Randomization will be conducted in blocks of four weeks to avoid overlapping participants in both groups. Patients admitted over a four-week period will be assigned to a single group (IG or CG). There will be a one-week period without inclusion to discharge patients included in the previous weeks, and then another four-week block will begin to assign the next group.

INTERVENTIONS

Control Group (CG):

They will receive standard treatment, which includes protocols for delirium or functional decline prevention, with removal of physical and pharmacological barriers and daily mobilization.

Intervention Group (IG):

They will receive physical training (PT) and health education. The PT program will consist of muscle strengthening exercises, balance training, gait stimulation, and inspiratory muscle training. The duration and intensity will be adjusted to the clinical situation and patient availability. Ideally, two PT sessions will be conducted daily (one in the morning and one in the afternoon), with an average duration of 30 minutes each, from Monday to Friday.

Warm-up: Each session will begin and end with a low-intensity warm-up and cool-down period of approximately 3 minutes, during which joint mobility exercises and stretching of major muscle groups will be performed.

Muscle Strengthening ("squats"): The muscle strengthening exercises will consist of a circuit primarily targeting the lower limbs (1-3 sets of 3-10 repetitions), alternating with exercises for the upper limbs (1-2 sets of 3-10 repetitions). They will be performed in a seated position on a chair with armrests, initially with both feet fully supported on the floor (knees flexed at 90°) and the back straight against the backrest (hip flexed at 90°). Starting from the initial position, participants will have to stand up from the chair and sit back down.

Difficulty Levels (not progressing to the next difficulty level until the patient completes 3 sets of 10 repetitions at the starting difficulty level):

1. With assistance, with arm support, without additional load, and with both feet supported.
2. With assistance, without arm support, without additional load, and with both feet supported.
3. Without assistance, without arm support, without additional load, and with both feet supported.
4. Without assistance, without arm support, with additional load, and with both feet supported (progress to the next difficulty level when reaching the maximum load allowed by weighted vests).
5. Without assistance, without arm support, without additional load, and with single-foot support.
6. Without assistance, without arm support, with additional load, and with single-foot support.

Balance: In a standing position, the patient will have to lift one knee for a certain period of time, progressing from 3 to 5 seconds. Subsequently, they will maintain balance by performing the same exercise with their eyes closed, following the same time progression. Up to 3 repetitions will be performed with each leg alternately. To stabilize the patient and provide safety, they will be held by the hands, while providing only necessary assistance.

Gait Stimulation: Patients who can walk with or without assistance will perform a gait stimulation exercise, which consists of walking as much as possible, with or without aid, for 10 minutes. The exercise will always be supervised, with the participant accompanied throughout the course. Participants should be able to maintain a conversation without difficulty breathing while walking. The time (up to a maximum of 10 minutes) and distance covered (according to marks placed in the hallway) will be recorded. When they are able to walk continuously for 10 minutes, the speed of movement will be increased.

Cool-down: Stretching exercises for major muscle groups will be performed, always adapted to individual characteristics of the participants.

Inspiratory Exercises: Participants will perform 30 inspirations through a device provided by the team (Power Breathe© Classic Medium Resistance) against 40% of maximum inspiratory pressure (PImax). Every 5 sessions, participants' PImax will be reassessed to adjust the workload.

Health Education: In each activity session, patients and caregivers will be taught how to perform the exercises to ensure they continue doing them at home, and prior to discharge, an entire session will be dedicated to reviewing the complete program. Essentially, the type, frequency, and progression of exercises to be performed will be reviewed. Participants will be explained how to perform them at home, and personalized written instructions with illustrations of the exercises will be provided. Additionally, one

month and two months after discharge, the professional who conducted the training will make a phone call to emphasize program adherence or address any doubts.

DATA SOURCE

Data will be obtained through interviews and review of medical records. Upon admission, each patient, family member or caregiver, and the responsible nurse will be interviewed. The patient and nurse will also be interviewed at hospital discharge, and a telephone contact will be made with the patient and family member or caregiver at 3 months post-discharge.

MEASUREMENT OF VARIABLES

Primary Variable:

The proportion of patients who, at 3 months post-hospital discharge, recover the Katz index they had two weeks before admission (baseline) will be measured. The Katz index (Katz S. JAMA 1963; 185:914-919) measures the number of Activities of Daily Living (ADLs) performed independently, including bathing, dressing, toileting, transferring from bed to chair, continence, and feeding. For each ADL, a score of 1 is given if performed independently and 0 if any assistance from another person is required. The range is from 0 to 6.

Measurement of Secondary Variables:

The Barthel Index assesses 10 items (feeding, bathing, dressing, grooming, bowel control, bladder control, toileting, transferring from bed to chair, ambulation, and stair climbing), with a range from 0 to 100 in 5-point intervals. A lower score indicates greater dependency (Mahoney F. Md Med J 1965; 14:61-65). The Functional Ambulation Classification (FAC) scale (Holden MK. Phys Ther 1984; 64:35-40) assesses walking ability in 6 levels: 0 unable to walk; 1 walks with substantial assistance from one person; 2 requires slight physical contact from one person; 3 walks independently but needs supervision from one person; 4 walks independently on level ground but cannot negotiate stairs; and 5 walks independently on level ground and stairs. These three scales (Katz, Barthel, and FAC) will be measured at 4 time points: 2 weeks before admission (baseline), at admission, at discharge, and at 3 months. The Short Physical Performance Battery (SPPB) (Guralnik JM. J Gerontol 1994; 49:M85-94) assesses the ability to perform three tasks (balance, gait speed, and chair stands) with a total score ranging from 0 to 12. Balance is evaluated by having the participant stand for ten seconds in three different positions: feet together, semi-tandem, and tandem. One point is awarded for each successfully performed position, with a range from 0 to 4. Gait speed is assessed by timing the participant walking 4 meters with or without the use of a cane or walker. Scores range from 4 (less than 4.8 seconds) to 0 (unable to perform). Chair stands measure the time it takes to stand up from a chair five times as quickly as possible without using the arms. Scores range from 4 (less than 11.2 seconds) to 0 (more than 60 seconds or unable to perform). These measures will be taken at admission and discharge. All outcome variables will be assessed by the same properly trained investigator who is not involved in the intervention. An investigator who did not participate in the training will conduct a telephone follow-up at three months post-discharge with the participant or caregiver (from both groups) to assess the functional status of ADLs (Katz index, Barthel index) and ambulation (FAC).

Other Variables:

They will be collected through a standardized questionnaire administered by trained personnel, including: Demographic data. Medical variables: cause of hospitalization and severity (Apache II scale (Crit Care Med 1985; 13:818)), comorbidity (Charlson index (Charlson ME. J Chronic Dis 1987; 40:373-383)), medication use (statins, psychotropics, cholinesterase inhibitors, corticosteroids, diuretics, and cardiovascular medications), history of falls, presence of geriatric syndromes (malnutrition, dementia, pressure ulcers, depression, delirium) diagnosed based on clinical criteria and commonly used scales, and presence of frailty, using Fried's criteria (Fried L, J Gerontol Med Sci 2001; 56A:M146-M156). Body

mass index (BMI), calculated as weight in kilograms divided by height in meters squared. Metabolites in urine.

SAMPLE SIZE

Calculated by estimating the proportion of patients who recover their baseline Katz index at 3 months post-hospital discharge. In our AGECAR study, in the control group, 40% of patients recovered their baseline Katz index at 3 months. With the current intervention, we expect that 60% will recover the Katz index at 3 months. Therefore, with a statistical power (1-β) of 80% and a significance level (α) of 0.05 (two-tailed), we need to analyze a total of 188 patients, rounded to the nearest "10," which is 190 patients. Hospital mortality in our unit is approximately 12%, and assuming a 20% loss to follow-up, we will need to include 252 patients, 126 in the intervention group and 126 in the control group.

FAMILIARIZATION AND RELIABILITY ASSESSMENT

Before the study begins, all subjects will have familiarization sessions lasting about 30 minutes, consisting of an explanation of the study objectives and the performance of all tests. Test-retest reliability will be determined for each measurement of the primary variable in a subpopulation of subjects.

ASSESSMENT OF ADVERSE EFFECTS (AEs)

Objectified or participant-reported AEs, including muscle pain, fatigue, and nonspecific pain, will be recorded by study personnel. Falls during the study period will also be assessed. An independent investigator will review all medical and nursing records to detect any falls.

PARTICIPATION AND ADHERENCE

The expected benefits will be explained to participants in the intervention group to reduce dropouts and maintain good adherence to the program. The distribution of training sessions throughout the day will minimize fatigue and contribute to adherence. Adherence to training sessions will be monitored daily with specific records, as well as the reasons for non-completion of any sessions.

STATISTICAL ANALYSIS

Groups will be compared using Student's t-test for continuous variables (or its nonparametric equivalent, the Mann-Whitney test), and chi-square test for categorical variables. The effect of training on outcome variables will be analyzed according to the principle of "intention-to-treat." The effect of training on the primary outcome variable, the proportion of patients who recover the Katz index at 3 months, will be analyzed using univariate and multivariate logistic regression. The effect of training on the recovery of other functional status scales will be analyzed similarly. The change in functional status from admission to discharge and 3 months will be classified as improvement if the number of independently performed ADLs increases or deterioration if the number decreases. Differences between groups will be analyzed using the chi-square test for linear trend. The effect of training on these functional scales as continuous variables will be analyzed using repeated measures analysis of variance (ANOVA) with two factors (between-subjects group and within-subjects time). The consistency of differences between the intervention and usual care in subgroups defined by age, frailty, presence of delirium, and baseline functional status will be analyzed using multivariate logistic regression with normal and ordinal models.

REFERENCES

1. Abellán García A, Ayala García A, Pujol Rodríguez R. 2017. “Un perfil de las personas mayores en España, 2017. Indicadores estadísticos básicos”. Madrid, Informes Envejecimiento en red nº 15, 48 p. [Fecha de publicación: 31/01/2017; última versión: 27/03/2017]. Accesible en http://envejecimiento.csic.es/documentos/documentos/enred-indicadoresbasicos17.pdf

2. Fried TR, et al. Health outcome prioritization as a tool for decision making among older persons with multiple chronic conditions. Arch Inter Med 2011; 171:1854 -1856

3. Mudge AM, et al. Timing and risk factors for functional changes associated with medical hospitalization in older patients. J Gerontol Biol Sci Med Sci 2010; 65:866 -872

4. Oakland HT, et al. The effect of short, unplanned hospitalizations on older adult functional status. J Am Geriatr Soc 2014; 62:788 -789

5. Zisberg A, et al. Hospital-associated functional decline: the role of hospitalization processes beyond individual risk factors. J Am Geriatr Soc 2015; 63:55 -62

6. Zisberg A et al: Low Mobility during Hospitalization and Functional Decline in Older Adults. J Am Geriatr Soc 2011; 59:266-273.

7. Brown CJ, et al. Barriers to mobility during hospitalization from the perspectives of older patients and their nurses and physicians. J Hosp Med. 2007; 2:305-1

8. Kortebein P et al: Effect of 10 Days of Bed Rest on Skeletal Muscle in Healthy Older Adults. JAMA 2007; 297:1772-1774

9. Coker RH, et al. Bed rest promotes reductions in walking speed, functional parameters, and aerobic fitness in older, healthy adults. J Gerontol Biol Sci Med Sci 2014; 70:91 -96

10. Hvid LG, et al. Aging impairs the recovery in mechanical muscle function following 4 days of disuse. Exp Gerontol 2014; 52:1 -8

11. Covinsky KE, et al. Hospitalization-associated disability: "She was probably able to ambulate, but I'm not sure" JAMA 2011; 306:1782-93

12. Gill TM et al. Hospitalization, restricted activity, and the development of disability among older persons. JAMA 2004; 292:2115-24

13. McCusker J et al: Predictors of Functional Decline in Hospitalized Elderly Patients: A Systematic Review. J Gerontol A Biol Sci Med Sci 2002; 57:M569-77

14. Mudge AM et al: Timing and Risk Factors for Functional Changes Associated with Medical Hospitalization in Older Patients. J Gerontol A Biol Sci Med Sci 2010; 65:866-872

15. Boyd CM, et al. Recovery of activities of daily living in older adults after hospitalization for acute medical illness. J Am Geriatr Soc 2008; 56:2171-9

16. Sleiman I et al: Functional Trajectories during Hospitalization: A Prognostic Sign for Elderly Patients. J Gerontol A Biol Sci Med Sci 2009; 64:659-663

17. Villumsen M, et al. Very low levels of physical activity in older patients during hospitalization at an acute geriatric ward --a prospective cohort study. J Aging Physical Activity 2014; 23:542 -549

18. Pedersen MM, et al. Twenty-four-hour mobility during acute hospitalization in older medical patients. J Gerontol A Biol Sci Med Sci 2012; 68:331 -337

19. Brown CJ et al: Prevalence and Outcomes of Low Mobility in Hospitalized Older Patients. J Am Geriatr Soc 2004; 52:1263-70.

20. Landefeld CS, et al. A randomized trial of care in a hospital medical unit especially designed to improve the functional outcomes of acutely ill older patients. N Eng J Med 1995; 332:1338-1344

21. Cohen HJ, et al. A controlled trial of inpatients and outpatients geriatric evaluation and management. N Eng J Med 2002; 346:905-912

22. Vidán MT, et al. An Intervention Integrated into Daily Clinical Practice Reduces the Incidence of Delirium During Hospitalization in Elderly Patients. J Am Geriatr Soc 2009; 57: 2029-2036

23. Bachmann S et al: Inpatient Rehabilitation Specifically Designed for Geriatric Patients: Systematic Review and Meta-Analysis of Randomised Controlled Trials. BMJ 2010; 340:c1718

24. Kosse NM, et al. Efectiveness and feasibility of early physical rehabilitation programs for geriatric hospitalized patients: A systematic review. BMC Geriatrics 2013: 13:107

25. Fox MT, et al. Acute care for elders components of acute geriatric unit care: Systematic descriptive review. J Am Geriatr Soc 2013;61:939-46

26. Siebens H, et al. A randomized controlled trial of exercise to improve outcomes of acute hospitalization in older adults. J Am Geriatr Soc 2000; 48:1545-1552

27. Jones CT, et al. A randomised controlled trial of an exercise intervention to reduce functional decline and health service utilisation in the hospitalized elderly. Australas J Ageing 2006, 25:126 -33

28. Brown CJ, et al. Comparison of posthospitalization function and community mobility in hospital mobility program and usual care patients a randomized clinical trial. JAMA Intern Med 2016; 176:921-27

29. Serra-Rexach JA, et al. Short-Term, Light- to Moderate-Intensity Exercise Training Improves Leg Muscle Strength in the Oldest Old: A Randomized Controlled Trial. J Am Geriatr Soc. 2011; 59: 594-602

30. Fleck SJ, et al. Activity in GEriatric acute CARe (AGECAR): rationale, design and methods. BMCGeriatr 2012; 12:28
