# Supplementary material for: Exercise Intervention and Hospital-Associated Disability: A Nonrandomized Controlled Clinical Trial
Source: JAMA Netw Open. 2024 Feb 8;7(2):e2355103. doi: 10.1001/jamanetworkopen.2023.55103 (PMC10853827; doi:10.1001/jamanetworkopen.2023.55103)
Supplement: Supplement 2. — eMethods. Supplementary Methods eTable 1. Detailed Description of Exercise Adherence eTable 2. Ad Hoc Analysis With Multiple Imputation: Effects of the Intervention on Hospital-Associated Disability (HAD), Ambulatory Capacity Decline, Falls and Readmission Rates. eTable 3. Per Protocol Analysis of Participants’ Characteristics at the Start of the Study and Length of Hospitalization, by Group eTable 4. Per Protocol Analysis of the Effects of the Intervention on Hospital-Associated Disability (HAD), Ambulatory Capacity Decline, Falls and Readmission Rates eTable 5. Per Protocol Analysis of the Effects of the Intervention on Physical Performance After Hospitalization eReferences [file jamanetwopen-e2355103-s002.pdf]

Supplementary Online Content

Rodriguez-Lopez C, Mayordomo-Cava J, Zarralanga-Lasobras T, et al. Exercise intervention and hospital-associated disability: a nonrandomized controlled clinical trial. *JAMA Netw Open*. 2024;7(1):e2355103. doi:10.1001/jamanetworkopen.2023.55103

eMethods. Supplementary Methods

eTable 1. Detailed Description of Exercise Adherence

eTable 2. Ad Hoc Analysis With Multiple Imputation: Effects of the Intervention on Hospital-Associated Disability (HAD), Ambulatory Capacity Decline, Falls and Readmission Rates.

eTable 3. Per Protocol Analysis of Participants' Characteristics at the Start of the Study and Length of Hospitalization, by Group

eTable 4. Per Protocol Analysis of the Effects of the Intervention on Hospital-Associated Disability (HAD), Ambulatory Capacity Decline, Falls and Readmission Rates

eTable 5. Per Protocol Analysis of the Effects of the Intervention on Physical Performance After Hospitalization

eReferences

This supplementary material has been provided by the authors to give readers additional information about their work.

© 2024 Rodriguez-Lopez C et al. *JAMA Network Open.*

eMethods. Supplementary Methods

Description of usual care

Usual care was defined as the standard care provided by geriatricians, residents, nurses (including one specialist geriatric nurse and a trainee specialist geriatric nurse) and, when appropriate, ancillary staff (nutritionists, rehabilitation team, social workers) when needed, who usually worked in the geriatric ward. Standard care in the acute care for elders (ACE) unit is focused on the prevention and management of geriatric syndromes, including a multi-domain work routine that has proven effective in reducing the incidence of delirium and functional decline during hospitalization (for details, see Vidan et al.).[1] This work routine includes general and specific areas such as orientation, sensorial perception, sleep maintenance, hydration, nutrition, review of psychoactive and sedative medications, and mobilization. In terms of mobilization, the following actions are considered: I) getting patients out of bed every day during admission, II) avoiding continuous fluid therapy, III) removing urinary catheters if possible, IV) initiating mobilization in the room and in the ward corridors and reminding the patient to do so every day, V) changing the position in bed every 3 hours if mobilization is not possible, and VI) avoiding physical restraints (used only with medical authorization and signature of physician).

Description of the exercise intervention

The Consensus on Exercise Reporting Template (CERT) statement was followed to describe the exercise intervention,[2] as follows. In-hospital exercise sessions were planned to last from the beginning to the end of the entire stay in the ACE unit, under the supervision of exercise instructors (a physiotherapist and a clinical exercise physiologist) and performed individually in the patient's room and in the ward corridor.

Exercise sessions included muscle strength, balance and aerobic training followed by inspiratory muscle training, and took place twice a day (morning and afternoon) except for weekends. Exercise sessions began with a brief seated **warm-up** consisting of active joint-specific mobilization (i.e., ankle, knee, shoulder, elbow, wrist, and neck) followed by **muscle strength exercises**.

Participants completed 3 sets of up to 15 repetitions of seated chest presses, lateral pull-downs and horizontal rows using elastic bands (Theraband, OH) with progressively increasing

© 2024 Rodriguez-Lopez C et al. *JAMA Network Open.*

levels of resistance (from 1.3 to 2.1 kg), followed by 3 sets of up to 10 repetitions of chair stands (using an armrest or assistance if needed). Rest periods of up to 2 minutes were allowed between sets and exercises at the patient's request. Individual starting levels were established according to patient capacity based on the elastic band resistance level for upper-body exercises or on the level of assistance required to complete at least half of the sets and repetitions for the remaining exercises. Intensity progression for each exercise was achieved by increasing the elastic band resistance or by reducing the level of assistance when patients reached the highest range of set and repetitions.

Strength exercises were followed by **balance training**, where the participants were asked to stand on one leg (with assistance if needed) for 3-5 seconds, completing up to 3 repetitions with each leg, and then repeat the same task with their eyes closed. Patients then performed the **aerobic component of the training**, which consisted of walking around the ward for up to 10 minutes. They were asked to walk faster than their usual walking speed, such that it was difficult to maintain a fluid conversation while still being able to speak. Assistance (mobility aids or an external person) was provided as needed, and short resting periods (30 seconds) were included if the required intensity could not be maintained.

Finally, we considered it was important to include **inspiratory muscle training** in our multicomponent exercise program because, in addition to the normal age-related decline in lung function (e.g., increase in chest wall stiffness, decrease in respiratory muscle strength and in alveolar surface area), supine position during bed rest may compromise lung function due to restricted chest wall mobility and diaphragmatic movement.[3,4] Moreover, bed rest is associated with respiratory muscle atrophy, predisposing individuals to ventilation-perfusion mismatch (i.e., impaired gas exchange), and increased risk of atelectasis and respiratory infections (i.e., caused by decreased cough effectiveness and increased risk of aspiration).[5] Under these circumstances, hospitalized older adults are at increased risk of respiratory complications, prolonged hospital stays, dyspnea and exercise intolerance,[6] which promote physical inactivity and increase the risk of developing hospital-associated disability.

While physical activity, and particularly aerobic exercise is beneficial for lung function,[3] targeting the respiratory muscles through inspiratory muscle training is effective in increasing

© 2024 Rodriguez-Lopez C et al. *JAMA Network Open.*

pulmonary function in healthy older adults[7], and also in older patients with COPD, heart failure or inspiratory muscle weakness.[8,9] It can therefore be hypothesized that this strategy might also be effective in patients with reduced mobility, as their ability to perform sustained aerobic exercise at a moderate-to-vigorous intensity is inherently limited. In this program, inspiratory muscle training was performed using a threshold training device (Power Breathe © Classic Medium Resistance, Micro RPM Care Fusion, San Diego, CA) with the patient seated with a clip on the nose. The program consisted of 3 sets of 10 repetitions against a resistance set at 40% of basal maximal inspiratory pressure, with 1-minute rest between sets. Patients were instructed to exhale to residual volume, and then perform a maximal inspiration (i.e., as fast and for as long as possible) through the aforementioned device. Maximal inspiratory pressure was determined during the first training session and reassessed every 6 sessions using a specific device (CareFusion, Kent, UK) to adjust the training load accordingly.

Adherence to the exercise program was determined as the number of sessions completed divided by the number of sessions originally planned for each patient. **Adverse events** were recorded during each session by the researchers responsible for supervising the exercise.

Patients in the intervention group also received a **health education program** to promote exercise adherence after hospital discharge. At the end of each exercise session, the patients and their relatives or caregivers received an educational session by the physiotherapist to explain all the exercises until the patients were able to do them on their own and repeat them at home after hospital discharge—see also below. They were also given a diary to record each completed session to encourage adherence. At discharge, patients were given an inspiratory muscle training device and a simple booklet with instructions to follow a home-based exercise program (similar to the hospital intervention) for 3 months after discharge. During this period, the patients received **monthl**y **telephone counseling** from a physiotherapist to remind them to follow the exercise program and to record any adverse events related to the exercise sessions.

Costs and full-time equivalents assigned to the intervention.

To comprehensively evaluate human resource utilization and associated costs for the exercise intervention, a detailed full-time equivalent (FTE) calculation was conducted. This entailed quantifying the time expended by each staff member involved in delivering the exercise

© 2024 Rodriguez-Lopez C et al. *JAMA Network Open.*

program. The nursing and geriatrician staff of our ACE unit (i.e., assessment staff) dedicated approximately 10 minutes to informing patients and caregivers about the intervention and obtaining informed consent. The assessments at admission and discharge required 40 and 20 minutes, respectively, with an additional 15 minutes for the telephone interview at the 3-month follow-up. Based on a standard 40-hour work week, 0.04 FTE was allocated for the evaluation of each patient.

Conversely, physiotherapists conducted exercise and health education sessions lasting approximately 40 minutes per session, scheduled twice daily (excluding weekends). Given the median length of stay in our study was 7 days, a total of 10 sessions per patient should be planned. The health education session at discharge and the two post-discharge telephone counseling calls lasted approximately 20 and 15 minutes, respectively. Overall, the delivery of the exercise and health education program required 0.19 FTE of a physiotherapist per patient.

In our hospital, the annual salary costs for a specialized geriatric nurse and a geriatrician are approximately $43,500 and $71,000, respectively. For a physiotherapist, the annual cost is approximately $41,500. Factoring in the cost of individual exercise equipment (around $53) and the FTE of the corresponding staff, the estimated cost of the intervention ranged between $231 and $250 per patient.

Ad hoc analyses

Ad hoc analyses were conducted to assess the effects of the intervention on the primary and secondary outcomes. First, a multiple imputation procedure was used to address the missing data in the outcomes of ADL function, ambulatory capacity, and readmission and fall rates. Briefly, multiple imputation consisted of a regression-based procedure to generate multiple copies of the dataset, each containing different estimates of the missing values. Baseline characteristics and length of stay were used in the prediction model. Imputation was performed by predictive mean matching for continuous data and logistic regression for binary variables, with constraints set to control the imputed values (e.g., scale ranges). We generated 25 imputed datasets following the recommendation to use as many imputations as the percentage of observations with missing data (~25% for the primary outcome).[10] Statistical tests used in the primary analysis were performed on each of the imputed datasets and pooled according to Rubin's rules. Additionally, adjusted

© 2024 Rodriguez-Lopez C et al. *JAMA Network Open.*

analyses were performed using the baseline value (i.e., 2 weeks prior to admission) of the relevant outcome as a covariate, except for falls and readmission rates.

© 2024 Rodriguez-Lopez C et al. *JAMA Network Open.*

eTable 1. Detailed description of exercise adherence

	Morning session	Afternoon session	Total
In-hospital sessions	3[2]	2 [2]	5 [4]
Adherence, % [a]	66.7 [17.9]	33.3 [28.4]	50.0 17.2
Session total duration, minutes	34 [7]	28 [7]	32 [6]
Strength exercise adherence, % [b]	100 [0]	100 [0]	100 [0]
Total repetitions per exercise	30 [4]	30 [4]	30 [4]
Balance exercise adherence, % [b]	100 [0]	100 [0]	100 [0]
IMT adherence, % [b]	100 [0]	100 [0]	100 [0]
Total repetitions	23 [7]		
Pressure threshold, cm H_2O	13.6 [11.1]		
Aerobic exercise adherence,% [b]	100 [0]	100 [0]	100 [0]
Duration, minutes	6.8 [3.3]	7.3 [3.4]	6.8 [2.9]
Home exercise 3-month follow-up [c]			
Declared having exercised ≥15 days per month, %	NA	NA	75.0
Types of exercise performed:			
Chair rise, %	NA	NA	54.6
Balance, %	NA	NA	50.9
Walking, %	NA	NA	76.9
IMT, %	NA	NA	63.0
Participants completing the intervention as per protocol, %[e]	NA	NA	54.3

Abbreviations: IMT, inspiratory muscle training. Data are expressed as median [interquartile range], unless otherwise stated. Symbols: [a] adherence was determined as the number of sessions completed divided by the number of sessions originally planned for each patient; [b] data are expressed as percentage of completed sessions including the corresponding exercise component; [d] data are expressed as percentage of the 108 patients contacted at 3-month follow-up; [e] percentage of the 108 patients contacted at 3-month follow-up who completed ≥50% of the prescribed in-hospital and home exercise sessions.

© 2024 Rodriguez-Lopez C et al. *JAMA Network Open.*

eTable 2. Ad hoc analysis with multiple imputation: Effects of the intervention on hospital-associated disability (HAD), ambulatory capacity decline, falls and readmission rates.

	**Intervention (n=130), No. (%)**	**Control (n=130), No. (%)**	Unadjusted estimates OR (95% CI), p-value	Adjusted estimates [a] OR (95% CI), p-value
**Primary Outcome:** *HAD determined by Katz Index*				
At discharge	64 (49.2)	78 (60.3)	0.64 (0.39 to 1.05), *P*=.08	0.59 (0.35 to 1.00), ***P*=.05**
At 3-month follow-up	49 (37.7)	62 (48.1)	0.66 (0.39 to 1.13), *P*=.13	0.62 (0.35 to 1.08), *P*=.09
**Secondary Outcomes**				
HAD determined by Barthel Index				
At discharge	77 (59.1)	97 (74.7)	0.49 (0.28 to 0.85), ***P*=.01**	0.48 (0.27 to 0.83), ***P*=.009**
At 3-month follow-up	48 (37.2)	77 (58.9)	0.41 (0.25 to 0.69), ***P*=.001**	0.38 (0.22 to 0.65), ***P*≤0.001**
Ambulatory capacity decline [b]:				
At discharge	37 (28.8)	52 (40.3)	0.60 (0.35 to 1.02), *P*=.06	0.57 (0.33 to 0.98), ***P*=0.04**
At 3-month follow-up	38 (29.1)	52 (40.3)	0.61 (0.34 to 1.11), *P*=.10	0.57 (0.31 to 1.06), *P*=0.08
Readmission[c]	42 (32.3)	59 (45.4)	IRR 0.76 (0.52 to 1.09), *P*=.09	NA
Falls[c]	28 (21.5)	36 (27.7)	IRR 0.70 (0.44 to 1.11), *P*=.11	NA

Significant p-values (p<0.05) are in bold. Abbreviations: CI, confidence interval; OR, odds ratio, IRR: incidence rate ratio, HR: hazard ratio. Symbols: [a] adjusted by baseline value (i.e., 2 weeks before admission) of the relevant outcome; [b] ambulatory capacity decline considered in the event of a decline in Functional Ambulatory Classification[11]; [c] data correspond to the number of patients with the event (percentage) during the 3-month follow-up, a total of 66 readmissions were registered for the intervention group and 86 for the control group, while a total of 34 falls were registered in the intervention group and 49 in the control group. The follow-up lasted up to 3 months after hospitalization [days of follow-up, median (IQR): 98 (11)]

© 2024 Rodriguez-Lopez C et al. *JAMA Network Open.*

eTable 3. Per-protocol analysis of participants' characteristics at the start of the study and length of hospitalization, by group.

**Variables**	**Intervention**[a] (n=59), No. (%)	**Control** (n=130), No. (%)
Age, mean (SD), years.	87.1 (4.7)	87.5 (5.0)
Women	25 (42.4)	76 (58.5)
BMI, mean (SD) $kg{\cdot}m^{-2}$ [b]	27.4 (4.6)	26 (4.5)
Living at home[c]	56 (94.9)	123 (95.3)
**Comorbidities**		
High comorbidity[b,d]	28 (47.5)	71 (55.5)
Heart failure[b]	32 (54.2)	64 (50.0)
Diabetes mellitus[b]	20 (33.9)	44 (34.4)
Moderate-severe CKD[b]	12 (20.3)	36 (28.1)
COPD[b]	20 (33.9)	31 (24.2)
Stroke[b]	9 (15.3)	24 (18.8)
Myocardial infarction[b]	13 (22.0)	22 (17.2)
Cancer[b]	10 (16.9)	14 (10.9)
Dementia[b]	5 (8.5)	20 (15.6)
**Geriatric Syndromes**		
Frailty phenotype[e]	31 (53.4)	80 (64.0)
Depression[b]	18 (30.5)	44 (34.1)
Falls[b]	17 (28.8)	41 (31.8)
Pressure ulcers[c]	1 (1.7)	3 (2.3)
Polypharmacy (≥7 drugs)[b]	43 (72.9)	94 (72.9)
MNA-SF screening score, mean (SD) [g]	11.19 (2.3)	10.3 (2.4)
Malnutrition risk	22 (38.6)	56 (49.1)
Confirmed malnutrition	4 (7.0)	18 (15.8)
**Main admission diagnosis** [g]		
Circulatory	15 (25.4)	36 (28.8)
Infection	13 (22.0)	24 (19.2)
Digestive	6 (10.2)	16 (12.8)
Respiratory	10 (16.9)	8 (6.4)
Blood/myeloproliferative disease	5 (8.5)	12 (9.6)
**Functional capacity**		
At baseline (2 weeks before admission)		
Katz Index, mean (SD) [h]	4.4 (1.7)	4.4 (1.7)
Independent ambulation[i]	43 (72.9)	88 (67.7)
At admission		
Katz Index, mean (SD) [j]	3.46 (2.0)	2.8 (1.9)
Independent ambulation[I,k]	30 (52.6)	50 (40.0)
**Length of stay**, median (IQR), days	7 (4)	6 (4)

Abbreviations: BMI, body mass index; CKD, chronic kidney disease; COPD, chronic obstructive pulmonary disease; IQR, interquartile range; MNA-SF, Mini nutritional assessment short form; SD, standard deviation. Symbols: [a] data correspond to 59 patients in the intervention group who completed ≥50% of the prescribed in-hospital and home exercise sessions, [b] data missing for 2 patients in the control group; [c] data missing for 1 patient in the control group; [d] high comorbidity defined as having a Charlson Comorbidity Index ≥3[12]; [e] frailty defined as having ≥3 of 5 Fried's criteria[13]; [f] MNA-SF, Mini-nutritional assessment-short form (ranging from 0 [worse] to 14 [best])[14], data missing for 18 patients (16 in the control group and 2 in the intervention group); [g] data missing for 5 patients in the control group; [h] higher scores indicate better function; [i] Independent ambulation considered if Functional Ambulatory Classification ≥4[11]; [j] data missing for 3 patients in the control group; [k] data missing for 5 patients in the control group.

© 2024 Rodriguez-Lopez C et al. *JAMA Network Open.*

eTable 4. Per-protocol analysis of the effects of the intervention on hospital-associated disability (HAD), ambulatory capacity decline, falls and readmission rates.

	**Intervention (n=59)[ab]**	**Control (n=130)[a]**	Unadjusted estimate OR (95% CI)	p-value
**Primary Outcome:** *HAD determined by Katz Index*				
At discharge	20/57 (35.1)	68/115 (59.1)	0.37 (0.19 to 0.72)	**.003**
At 3-month follow-up	14/57 (24.6)	39/93 (41.9)	0.45 (0.22 to 0.94)	**.03**
**Secondary Outcomes**				
HAD determined by Barthel Index				
At discharge	24/55 (43.6)	83/112 (74.1)	0.27 (0.14 to 0.53)	**<.001**
At 3-month follow-up	11/55 (20.0)	48/92 (52.2)	0.23 (0.11 to 0.5)	**<.001**
Ambulatory capacity decline [c]				
At discharge	13/59 (22.0)	44/115 (38.3)	0.46 (0.22 to 0.94)	**.03**
At 3-month follow-up.	7/58 (12.1)	30/93 (32.3)	0.29 (0.12 to 0.71)	**.007**
Readmission [d]	16/58 (27.6)	36/93 (39.6)	IRR 0.68 (0.39 to 1.18)	.171
Falls[d]	7/58 (12.1)	18/90 (20.0)	IRR 0.48 (0.19 to 1.24)	.130

Significant p-values (p<0.05) are in bold. Abbreviations: OR, odds ratio, CI, confidence interval; IRR: incidence rate ratio. Symbols: [a] data are presented as number of cases/total observations (percentage) unless otherwise indicated; [b] data correspond to 59 patients in the intervention group who completed ≥50% of the prescribed in-hospital and home exercise sessions; [c] ambulatory capacity decline considered in the event of a decline in Functional Ambulatory Classification[11]; [d] data correspond to the number of patients with the event/total patients observed (percentage) during the 3-month follow-up, a total of 22 readmissions were registered for the intervention group and 51 in the control group while a total of 9 falls were registered in the intervention group and 29 in the control group.

© 2024 Rodriguez-Lopez C et al. *JAMA Network Open.*

eTable 5. Per-protocol analysis of the effects of the intervention on physical performance after hospitalization.

	Intervention (n=59)[a]		Control (n=130)		
	Admission, mean (95% CI)	Change at discharge, mean (95% CI)	Admission, mean (95% CI)	Change at discharge, mean (95% CI)	Intervention effect (p-value)[b]
**SPPB[c]**					
Total score (0–12)	4.5 (4 to 5)	0.6 (0.2 to 1.1)	3.7 (3.2 to 4.1)	0.3 (0 to 0.6)	.28
Balance score (0–4)	2 (1.7 to 2.3)	0.2 (0 to 0.5)	1.5 (1.3 to 1.7)	0.2 (0 to 0.4)	.60
Gait score (0–4)	2.4 (2.1 to 2.7)	0.4 (0.2 to 0.6)	1.7 (1.5 to 1.9)	0.1 (-0.1 to 0.2)	**.01**
Gait speed, $m \cdot s^{-1}$	0.5 (0.5 to 0.6)	0.1 (0 to 0.1)	0.5 (0.4 to 0.5)	0 (0 to 0)	**.00**
5STS, score (0–4)	1 (0.7 to 1.2)	0 (-0.2 to 0.2)	0.6 (0.5 to 0.8)	0 (-0.1 to 0.2)	.83
5STS performance, s	15.3 (8.6 to 22)	0.6 (-8.5 to 9.7)	13.6 (9.1 to 18.1)	-2 (-8.4 to 4.4)	.65
**Alusti Test[d]**					
Total score (0–100)	64 (60.4 to 67.6)	3.7 (0.8 to 6.6)	61.6 (59.2 to 64)	-0.1 (-2.1 to 1.8)	**.034**
Joint mobility (0-8)	25.8 (25 to 26.7)	-0.2 (-1 to 0.6)	25.4 (24.8 to 25.9)	-0.3 (-0.9 to 0.3)	.902
Transfer and stability (0–20)	15.9 (14.7 to 17)	1.4 (0.4 to 2.4)	15.1 (14.3 to 15.9)	0.7 (0 to 1.5)	.303
Gait (0–32)	22 (20 to 24.1)	2.4 (0.6 to 4.1)	20.1 (18.8 to 21.5)	0.4 (-0.9 to 1.6)	.069
Balance (0-20)	3.6 (1.4 to 5.8)	-1.5 (-3.9 to 0.9)	2.8 (1.3 to 4.2)	-0.9 (-2.9 to 1.1)	.687

All data were derived from linear mixed-effects model. Abbreviations: 5STS, five times sit-to-stand test; CI, confidence interval; SPPB, short physical performance battery. Values in parentheses after the variable names denote the range for each scale, all from worst to best score. Symbols: [a]data correspond to patients who completed >50% of the prescribed in-hospital sessions; [c]the effect of the intervention was determined with the time-by-group interaction p-value; [c] a total of 187 patients were considered in the analysis of SPPB differences; [d] a total of 185 were considered in the analysis of Alusti Test differences.

© 2024 Rodriguez-Lopez C et al. *JAMA Network Open.*

eReferences

1. Vidán MT, Sánchez E, Alonso M, Montero B, Ortiz J, Serra JA. An intervention integrated into daily clinical practice reduces the incidence of delirium during hospitalization in elderly patients. *Journal of the American Geriatrics Society*. Nov 2009;57(11):2029-36. doi:10.1111/j.1532-5415.2009.02485.x
2. Slade SC, Dionne CE, Underwood M, Buchbinder R. Consensus on Exercise Reporting Template (CERT): Explanation and Elaboration Statement. *Br J Sports Med*. Dec 2016;50(23):1428-1437. doi:10.1136/bjsports-2016-096651
3. Roman MA, Rossiter HB, Casaburi R. Exercise, ageing and the lung. *Eur Respir J*. Nov 2016;48(5):1471-1486. doi:10.1183/13993003.00347-2016
4. Katz S, Arish N, Rokach A, Zaltzman Y, Marcus E-L. The effect of body position on pulmonary function: a systematic review. *BMC Pulm Med*. 2018/10/11 2018;18(1):159. doi:10.1186/s12890-018-0723-4
5. Okazaki T, Suzukamo Y, Miyatake M, et al. Respiratory Muscle Weakness as a Risk Factor for Pneumonia in Older People. *Gerontology*. 2021;67(5):581-590. doi:10.1159/000514007
6. Harper CM, Lyles YM. Physiology and Complications of Bed Rest. 1988;36(11):1047-1054. doi:https://doi.org/10.1111/j.1532-5415.1988.tb04375.x
7. Aznar-Lain S, Webster AL, Cañete S, et al. Effects of inspiratory muscle training on exercise capacity and spontaneous physical activity in elderly subjects: a randomized controlled pilot trial. *Int J Sports Med*. Dec 2007;28(12):1025-9. doi:10.1055/s-2007-965077
8. Seixas MB, Almeida LB, Trevizan PF, et al. Effects of Inspiratory Muscle Training in Older Adults. *Respir Care*. Apr 2020;65(4):535-544. doi:10.4187/respcare.06945
9. Manifield J, Winnard A, Hume E, et al. Inspiratory muscle training for improving inspiratory muscle strength and functional capacity in older adults: a systematic review and meta-analysis. *Age and ageing*. May 5 2021;50(3):716-724. doi:10.1093/ageing/afaa221
10. White IR, Royston P, Wood AM. Multiple imputation using chained equations: Issues and guidance for practice. *Stat Med*. Feb 20 2011;30(4):377-99. doi:10.1002/sim.4067
11. Holden MK, Gill KM, Magliozzi MR, Nathan J, Piehl-Baker L. Clinical gait assessment in the neurologically impaired. Reliability and meaningfulness. *Physical therapy*. Jan 1984;64(1):35-40. doi:10.1093/ptj/64.1.35
12. Charlson ME, Pompei P, Ales KL, MacKenzie CR. A new method of classifying prognostic comorbidity in longitudinal studies: development and validation. *Journal of chronic diseases*. 1987;40(5):373-83. doi:10.1016/0021-9681(87)90171-8
13. Fried LP, Tangen CM, Walston J, et al. Frailty in older adults: evidence for a phenotype. *J Gerontol A Biol Sci Med Sci*. Mar 2001;56(3):M146-56. doi:10.1093/gerona/56.3.m146
14. Kaiser MJ, Bauer JM, Ramsch C, et al. Validation of the Mini Nutritional Assessment short-form (MNA-SF): a practical tool for identification of nutritional status. *The journal of nutrition, health & aging*. Nov 2009;13(9):782-8. doi:10.1007/s12603-009-0214-7

© 2024 Rodriguez-Lopez C et al. *JAMA Network Open.*
