# Supplementary material for: Exercise Intervention and Hospital-Associated Disability: A Nonrandomized Controlled Clinical Trial
Source: JAMA Netw Open. 2024 Feb 8;7(2):e2355103. doi: 10.1001/jamanetworkopen.2023.55103 (PMC10853827; doi:10.1001/jamanetworkopen.2023.55103)
Supplement: Supplement 3. — Data Sharing Statement [file jamanetwopen-e2355103-s003.pdf]

Data Sharing Statement

Rodriguez-Lopez. Exercise Intervention and Hospital-Associated Disability. *JAMA Netw Open*. Published February 08, 2024. doi:10.1001/jamanetworkopen.2023.55103

Data
Data available: Yes
Data types: Participant data with identifiers
How to access data: Data will be shared with investigator support after approval of a proposal and a signed data access agreement. All data requests should be submitted to the corresponding author for consideration (crlopez@salud.madrid.org).
When available: With publication

Supporting Documents
Document types: None

Additional Information
Who can access the data: Access to anonymized data may be granted to researchers following review.
Types of analyses: Data will only be available for related research purposes, such as individual patient data meta-analyses.
Mechanisms of data availability: Data will be shared with investigator support after approval of a proposal and a signed data access agreement.
